# Discovery Potent of Thiazolidinedione Derivatives as Antioxidant, α-Amylase Inhibitor, and Antidiabetic Agent

**DOI:** 10.3390/biomedicines10010024

**Published:** 2021-12-23

**Authors:** Manal Y. Sameeh, Manal M. Khowdiary, Hisham S. Nassar, Mahmoud M. Abdelall, Suliman A. Alderhami, Ahmed A. Elhenawy

**Affiliations:** 1 Chemistry Department, Faculty of Applied Science, Alleeth University Collage, Umm Al-Qura University, Makkah 24211, Saudi Arabia; mmkhowdiary@uqu.edu.sa; 2 Applied Surfactant Laboratory, Egyptian Petroleum Research Institute, Nasr City 11727, Cairo, Egypt; 3 Department of Chemistry, Faculty of Science and Arts in Al-Mukhwah, Al-Baha University, Al-Mukhwah 65311, Saudi Arabia; hishamsan@bu.edu.sa (H.S.N.); Alderhami@bu.edu.sa (S.A.A.); 4 Chemistry Department, Faculty of Science, Al-Azhar University, Nasr City 11884, Cairo, Egypt; mohmoed@azhar.edu.eg

**Keywords:** thiazolidinedione, anti-diabetic, antioxidant, molecular docking

## Abstract

This work aimed to synthesize safe antihyperglycemic derivatives bearing thiazolidinedione fragment based on spectral data. The DFT theory discussed the frontier molecular orbitals (FMOs), chemical reactivity of compounds, and molecular electrostatic potential (MEP) to explain interaction between thiazolidinediones and the biological receptor. α-amylase is known as the initiator-hydrolysis of the of polysaccharides; therefore, developing α-amylase inhibitors can open the way for a potential diabetes mellitus drug. The molecular docking simulation was performed into the active site of PPAR-γ and α-amylase. We evaluated in vitro α-amylase’s potency and radical scavenging ability. The compound **6** has the highest potency against α-amylase and radical scavenging compared to the reference drug and other members. They have been applied against anti-diabetic and anti-hyperlipidemic activity (in vivo) based on an alloxan-induced diabetic rat model during a 30-day treatment protocol. The most potent anti hyperglycemic members are **6** and **11** with reduction percentage of blood glucose level by 69.55% and 66.95%, respectively; compared with the normal control. Other members exhibited moderate to low anti-diabetic potency. All compounds showed a normal value against the tested biochemical parameters (CH, LDL, and HDL). The ADMET profile showed good oral bioavailability without any observed carcinogenesis effect.

## 1. Introduction

Diabetes mellitus (DM) is a common chronic health problem, with about 416 million people worldwide suffering from DM. This figure is expected to increase by 2040 to 618 million cases [1,2,3,4]. The DM is classified into two major classes: as “DMI” and “DMII” [5,6]. About >90% of individuals suffer from DMII (non-insulin-dependent diabetes mellitus; “NIDDM”). The researchers recognized DMII by tissue resistance to the action of insulin combined with a reduction in insulin secretion due to resistance or deficiency regarding beta cells [6].

An increased blood glucose level is linked to raising several health problems, including cardiovascular disease, further insulin damage, and so one. DMII patients have a higher risk of developing cardiovascular disease than non-DM patients [7]. α-glucosidase and α-amylase are vital enzymes in the therapy process [8], and responsible for breakdown of α-D-(1,4)-glycosidic bond in starch to get monosaccharides [9]. The inhibition of these enzymes offers an effective protocol for reducing blood glucose level [7,10]. PPAR-γ(peroxisome proliferator-activated receptor) is an essential component in adipogenesis. It also serves a vital function via sensitivity to insulin, organization of the cell cycle, and cell-differentiation. The nuclear receptor family (NR1C3) includes PPAR-γ known as the glitazone receptor, which organizes the gene expression involved in glucose metabolism [11]. The clinical study showed that a mixed therapy involving α-glucosidase inhibitors and PPAR-γ-agonists [12,13,14] was a helpful strategy for the treatment of DMII, as acarbose mixed with pioglitazone. Acarbose prevents NIDDM, and reduces cardiovascular problem [15]. While pioglitazone decreases cardiovascular action and anti-atherosclerosis [16]. The pre-clinical results of rosiglitazone and pioglitazone revealed no negative effects throughout the short periods of therapy (30 or 48 days), but did exhibit side effects after prolonged periods of use (years). Thus, in this work, we hope to synthesize new compounds without or with low toxicity when applied as antidiabetic medication. However, future trials with prolonged compounds consumption will be conducted, and the results will be published.

**Figure 1:**
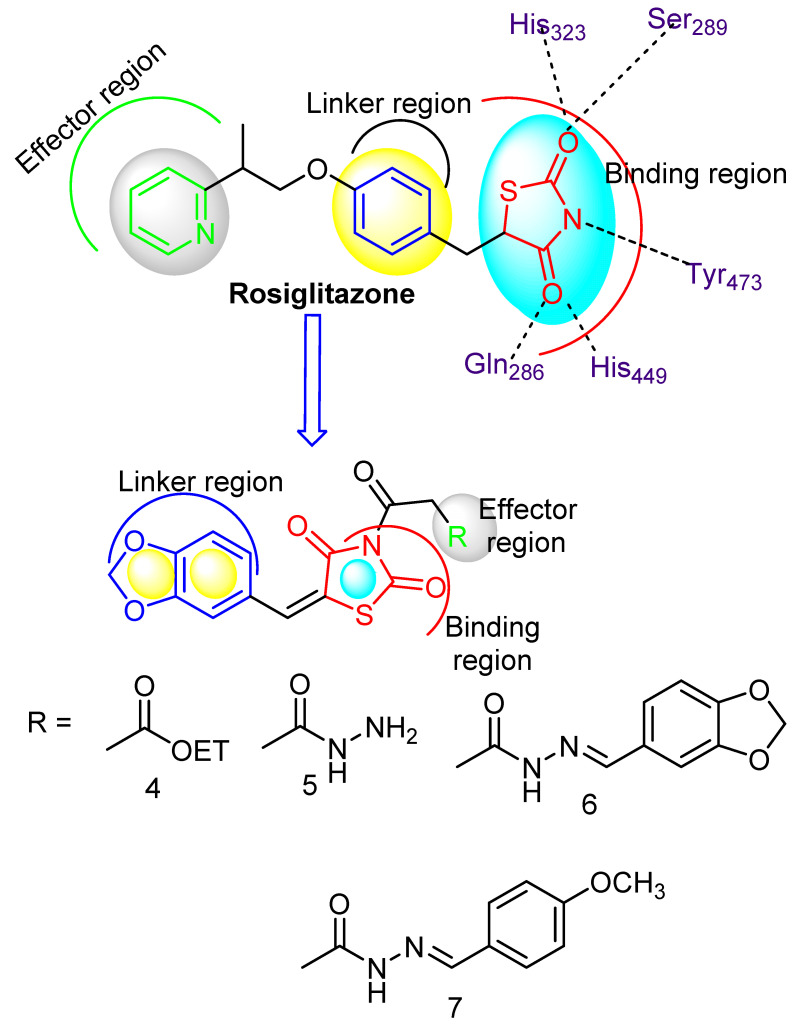
Designed molecule compliance of rosiglitazone pharmacophore.

Thiazolidinediones (TZDs) are an efficient DMII drug [17], (Figure 1). The combination therapy is also efficient for older patients that have hypertension, DMII [18], and induced-electrolyte-disturbance in rats with DMII [19]. Despite the efficiency of the drugs currently in use, such as acarbose and TZDs, they are associated with adverse side effects, such as diarrhea and obesity. The investigation of a more effective novel drug with low side effects is a valuable target [20]. Recently, uni-molecular and multi-target drugs have been developed to treat metabolic-syndrome, such as the inhibitor of Na-dependent-glucose-transporters “SGLTI” [21,22].

TZDs are hepatotoxic and carcinogenic, as well as being associated with dangerous side effects, such as oedema, obesity, and cardiac problems [23,24]. The 2,4-thiazolidinediones have several potential profiles against anticancer [25], anti-oxidant [26], anti-malarial [27], anti-obesity, and anti-microbial activities [27,28].

In view of the above mentioned facts, we reported the discovery of potent new synthetic thiazolidinedione derivatives as α-amylase inhibitors and antioxidants in vitro, and evaluation of in vivo anti-diabetic and antihyperlipidemic activity. We performed docking study also to gain further insights concerning the possible binding modes of our compounds.

## 2. Materials and Methods

Melting points, thin layer chromatography (R_f_), IR, NMR, elemental analyses (C, H, N), and mass spectra were recorded (Supplementary Figure S1). This study obeyed to standards of the ethics committee for the National Research center which complies with the National Regulations on Animal Welfare and Institutional Animal Ethical Committee (IAEC).

### 2.1. Chemistry

#### 2.1.1. Interaction of the Potassium Salt 3 with Halogenated Compounds

The proper halogenated compounds as chloroacetone and chloroacetic acid (0.1 mol) were refluxed with **3** (0.1 mol) in DMF (50 mL.) to give **5**, **6**, respectively. The crude products were separated and crystallized from the appropriate solvent.

*5-(benzo[d][1,3]dioxol-5-ylmethylene)-3-(2-oxopropyl) thiazolidine-2,4-dione* (**5**). Pale yellow crystals, yield 67%, m.p. 171–173 °C. IR (KBr): (ν/cm^−1^) = 1691, 1737 (CO). ^1^H-NMR (DMSO-d^6^) δ = 7.79 (s, 1H, CH = benzylidine), 7.20–7.14 (m, 1H-ArH), 7.10 (dd, *J* = 1.9, 0.5 Hz, 1H, ArH), 7.02 (d, *J* = 8.9 Hz, 1H, ArH), 6.01 (s, 2H, CH_2_ dioxole), 4.59 (s, 2H), 2.18 (s, 3H). ^13^C NMR (DMSO-d_6_) δ 201.41, 170.00, 168.67, 149.34, 148.33, 130.68, 128.12, 125.32, 119.76, 110.52, 109.65, 101.85, 48.74, 27.10. Anal. Calcd for C_14_H_11_NO_5_S (305): C, 55.08; H, 3.60; N, 4.59. Found: C, 55.03; H, 3.56; N, 4.53.

*2-(5-(benzo[d][1,3]dioxol-5-ylmethylene)-2,4-dioxothiazolidin-3-yl)acetic acid* (**6**). Yellow crystals, yield 71%, m.p. 188–190 °C. IR (KBr): (ν/cm^−1^) = 3421 (OH), 1711, 1744 (C = O). ^1^H-NMR (DMSO-d_6_) δ 9.93 (s, 1H, OH), 7.81–7.77 (m, 1H, CH = benzylidine), 7.20–7.14 (m, 1H, Ar-H), 7.10 (dd, *J* = 1.9, 0.5 Hz, 1H, Ar-H), 7.02 (d, *J* = 8.9 Hz, 1H, Ar-H), 6.01 (s, 2H, CH_2_ dioxole), 4.65 (s, 2H). ^13^C NMR (DMSO-d_6_) 170.51, 170.14, 168.51, 149.34, 148.33, 130.72, 128.12, 125.32, 119.76, 110.52, 109.65, 101.85, 41.71. Anal. Calcd for C_13_H_9_NO_6_S (307): C, 50.81; H, 2.93; N, 4.56. Found: C, 50.77; H, 2.91; N, 4.51.

#### 2.1.2. 2-(5-(Benzo[d][1,3]dioxol-5-ylmethylene)-2,4-dioxothiazolidin-3-yl)-2-oxoethyl (5-Methyl-1,3,4-thiadiazol-2-yl) Carbamodithioate (**9**)

The carbon disulfide was added to 5-methyl-1,3,4-thiadiazol-2-amine (**7**; 0.01 mol.) in the potassium hydroxide solution to give the corresponding potassium salt (**8**). The compound (**4**; 0.01 mol) was added, then the reaction mixture was stirred for 3 h, then diluted with water. The precipitate was collected, and finally recrystallized from acetic acid to afford the compound **9** as yellow crystals, yield 69%, m.p. 261–262 °C. IR (KBr): (ν/cm^−1^) = 1698, 1742 (CO). 1H-NMR (DMSO-d6) δ 10.48 (s, 1H, NH), 7.99 (d, *J* = 0.5 Hz, 1H, CH = benzylidine), 7.20–7.14 (m, 1H, Ar-H), 7.10 (dd, *J* = 1.9, 0.5 Hz, 1H, Ar-H), 7.02 (d, *J* = 8.9 Hz, 1H, Ar-H), 6.01 (s, 2H,), 4.25 (s, 2H, CH2 dioxole), 2.79 (s, 3H). C13NMR (DMSO-d6) δ 199.55, 168.79, 167.44, 165.66, 163.42, 157.52, 149.34, 148.33, 130.57, 128.22, 125.32, 122.05, 110.52, 109.65, 101.85, 35.90, 15.18. Anal. Calcd for C_17_H_12_N_4_O_5_S_4_ (480): C, 42.50; H, 2.50; N, 11.66. Found: C, 42.45; H, 2.44; N, 11.62.

#### 2.1.3. 5-(Benzo[d][1,3]dioxol-5-ylmethylene)-3-(2-(2,4-dioxothiazolidin-3-yl)acetyl) Thiazolidine-2,4-dione (**11**)

The compound (**1**; 0.1 mol) was heated with (**4**; 0.1 mol) in DMF (30 mL) for 3 h. The reaction mixture was cooled, and the solid product was filtered off, dried, crystallized from DMF to give (**5**) as pale yellow crystals, yield 73%, m.p. > 300 °C. IR (KBr): (ν/cm^−1^) = 1696, 1740 (CO). ^1^H-NMR (DMSO-d_6_) δ 7.99 (d, *J* = 0.4 Hz, 1H, CH = benzylidine), 7.20–7.14 (m, 1H, Ar-H), 7.10 (dd, *J* = 1.9, 0.5 Hz, 1H, Ar-H), 7.02 (d, *J* = 8.9 Hz, 1H, Ar-H), 6.01 (s, 2H, dioxole ring), 4.96 (s, 2H), 3.82 (s, 2H, thiazoldinone). ^13^C NMR (DMSO-d^6^) δ 177.61, 172.56, 167.67, 166.89, 165.73, 149.34, 148.33, 130.68, 128.22, 125.32, 122.05, 110.52, 109.65, 101.85, 43.92, 31.65. Anal. Calcd. for C_16_H_10_N_2_O_7_S_2_ (406): C, 47.29; H, 2.46; N, 6.89. Found: C, 47.24; H, 2.42; N, 6.85.

### 2.2. Computational Study

#### 2.2.1. Preparation of the Small Molecule

The target compounds were built, and their energy was minimized with PM3 through MOPAC [29] then the DFT using B3LYP/6-311G * was applied (Supplementary Materials).

#### 2.2.2. Protein Selection and Molecular Docking

Docking experiment was carried out on the target active sites for *PPAR-**γ*** (ID: 2PRG) and α-amylase (ID: 2QV4) using MOE-2019. The inhibitors complexed with the crystal structures of *PPAR-**γ*** (ID: 2PRG) and α-amylase (ID: 2QV4) were obtained. Water and inhibitors were removed, then H-toms were supplemented by MOE-2019 suite tool, which was used to handle the kinase (pH = 7.4). All the ligands were fitted into the suitable binding sites. Formerly, the receptors were created with a parametric cubic-box (12 Å × 12 Å × 12Å) over a centroid-binding pocket. The other docking procedures are described in Supplementary Materials.

### 2.3. Biological Study

#### 2.3.1. Animals

Healthy male albino Wistar rats (150 ± 20 g) have been obtained as mentioned in Supplementary Materials.

#### 2.3.2. Induction of Diabetes

The animals were kept fasting overnight (12 h) before induction of diabetes, which was induced in rats as reported [30,31,32,33,34] and mentioned in Supplementary Materials.

#### 2.3.3. Determination of the Blood Glucose Levels

We obtained the blood samples from the tail vein of all rats, the blood-glucose-level “BGLs” were measured and monitored in all treatment groups at days 0 (basal), 2th, 4th, 15th, and 30th as reported [35].

#### 2.3.4. Biochemical Analysis

The blood samples (0.5 mL) were withdrawn from the retro-orbital plexus of each rat and the other steps adapted for measuring the biochemical analysis are shown in (Supplementary Materials).

#### 2.3.5. The In Vitro Inhibition of α-Amylase Assay

The α-amylase activity of the probe compounds determined using a method [36].

#### 2.3.6. DPPH Radical Scavenging Activity

The assay of 2,2-Diphenyl-1-picrylhydrazyl radical (DPPH) was performed according to the method at [37].

#### 2.3.7. Statistical Analysis

The analysis of the data is described in Supplementary Materials.

## 3. Results and Discussion

### 3.1. Chemistry

**Scheme 1:**
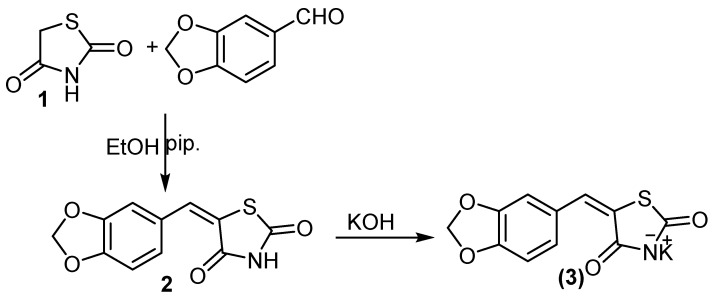
Synthesis of compounds **3**.

In a continuation of our previous work [37], we reported the synthesis of novel antidiabetic agents based on the thiazolidinedione scaffold. The 5-(benzo[d][1,3]dioxol-5-ylmethylene) thiazolidine-2,4-dione reacted with potassium hydroxide in DMF to give the potassium salt derivative (**3**), Scheme 1.

**Scheme 2:**
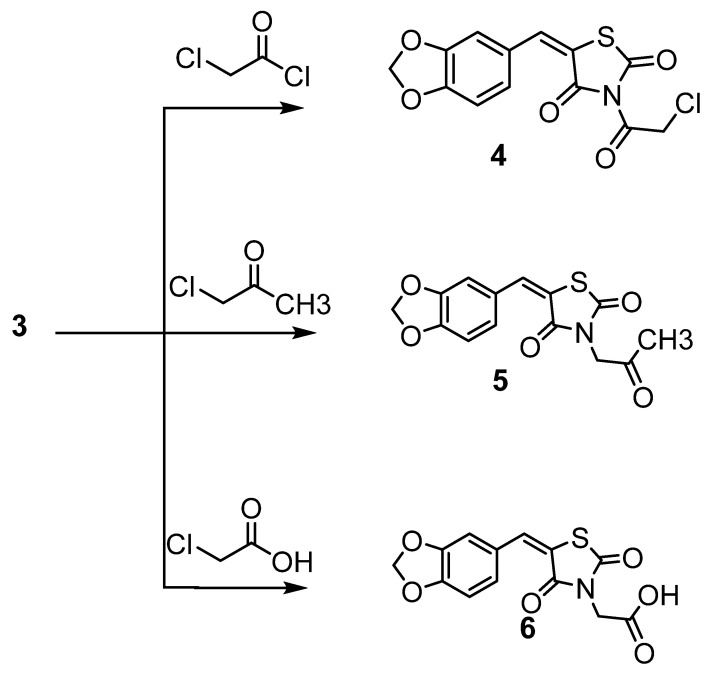
Synthesis of compounds **4–6**.

The molecule **3** underwent a further reaction with chloro-acetyl chloride to give chloroacetyl thiazolidine-2,4-dione **4** [37]. The interaction of the equimolar of potassium salt **3** with chloroacetone in DMF under a reflux condition afforded a 5-(benzo[d][1,3]dioxol-5-ylmethylene-3-(2-oxopropyl) thiazolidine-2,4-dione (**5**), Scheme 2. In the same manner, the potassium salt (**3**) reacted with chloroacetic acid to give 2-(5-(benzo[d][1,3]dioxol-5-ylmethylene)-2,4-dioxothiazolidin-3-yl) acetic acid (**6**) in good yield, Scheme 2.

**Scheme 3:**
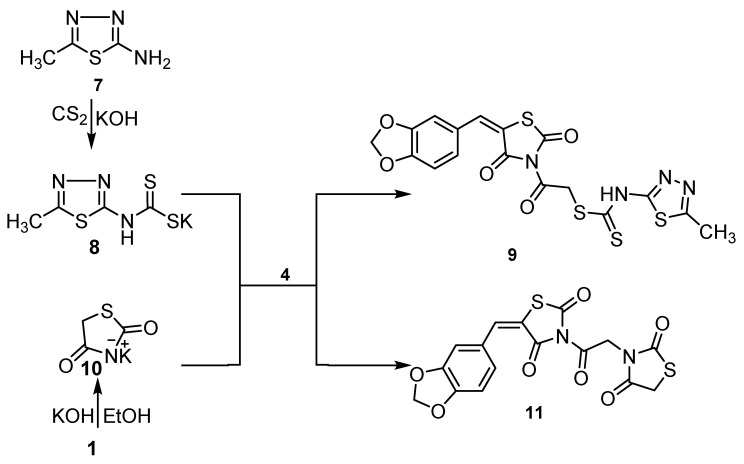
Synthesis of compounds **9** and **11**.

Compound (**4**) was heated with potassium (5-methyl-1,3,4-thiadiazol-2-yl)-carbamodithioate (**7**) in DMF under stirring condition to give 2-(5-(benzo[d][1,3]dioxol-5-ylmethylene)-2,4-dioxothiazolidin-3-yl)-2-oxoethyl (5-methyl-1,3,4-thiadiazol-2-yl)-carbamodithioate **9**, which displayed two absorption bands attributed to 2 C = O at 1698 and 1742 cm^−1^. The ^1^H NMR spectrum (DMSO-d_6_) of **9** revealed signals at δ = 2.79 (s, 3H, CH_3_), 3.60 (s,2H, CH_2_), 4.25 (s, 2H, CH_2_ of dioxole ring) and 10.48 (s, 1H, NH). Compound **4** interacted with the potassium 2,4-dioxothiazolidin-3-ide (**10**) to give 5-(benzo[d][1,3]dioxol-5-ylmethylene)-3-(2-(2,4-dioxothiazolidin-3-yl)acetyl)-thiazolidine-2,4-dione (**11**), Scheme 3. The IR spectrum (cm^−1^) of **11** revealed the absorption band at 1696 and 1740 cm^−1^ for the two (C = O) group. The ^1^H NMR spectrum (DMSO-d_6_) of **11** revealed signals at δ = 3.82 (s, 2H, CH_2_), 4.96 (s, 2H, CH_2_),6.01 (s, 2H, CH_2_ of dioxole ring), 7.99 (s, 1H, CH = benzylidene).

### 3.2. Molecular Modeling Studies

#### 3.2.1. Kinase Inter-/Intramolecular Interaction Stability Based on FMO Analysis

**Figure 2:**
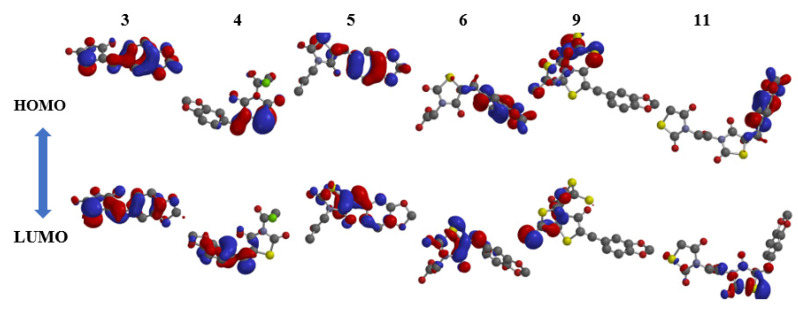
HOMO and LUMO density of compounds (**3**, **5**–**12**).

The DFT (density function theory) optimized the (**3**–**6**, **9**, **11**) hybrids using the B3YLP/6311G ** correlation function [38]. In all compounds, the thiazolidine-2,4-dione was stabilized with benzo[d][1,3]dioxole by planarity and/or coplanarity modes. The frontier molecular orbital “FMO” was defined as HOMO and LUMO (donating electron/accepting electron), respectively. These orbitals can decide the interaction rout with the kinase. FMO gap and chemical reactivity determined the kinetic stability of the molecule [39]. The increasing energy level of HOMO compounds reflected their potent ability for providing the electron and more susceptible to oxidation, and vice versa [40,41]. E_HOMO_ arranged the compounds in decreasing order **5** > **4** > **6** > **9** > **11** > **3** (Table 1). The HOMO sector is localized over thiazolidine-2,4-dione rings in compounds **3**–**5** and **9**. These compounds bear a LUMO area over benzodioxol ring. Inversely, the compound **11** has a HOMO area over benzodioxol and LUMO area covered thiazolidinedione (Figure 2).

The negative energy values for HOMO and LUMO indicated that the intramolecular charge transfer from the thiazolidine-2,4-dione to benzodioxole corners occurred in **3**–**6** and **9**. This direction was reversed in **11** molecule.

The energy gap ΔG is a vital parameter for examining the stability of the biomolecule in the receptor. ΔG is inversely linked with the binding interaction between ligand and receptor. The low value of ΔG for the compound postulated a good binding interaction between their FMOs and the receptor of the kinase [40].

We computed the chemical reactivity descriptors for the molecules in (Table 1), such as: S. softness (measures the stability of the molecule which direct proportional with chemical reactivity) [42]; η, hardness (the reciprocal of softness); χ, electronegativity strength [43]; μ^−^, the electron donation power; μ^+^, the potency for catching the electron; ω^−^, the electro-donation capacity; ω^+^, the electro-acceptance capacity; ω^±^, the net electrophilicity (measure the relative power between electron acceptance and electron donation) [44]; ωi, the electrophilicity index in the ground state (studding the decreasing energy got from the donor → acceptor movement) [45]. These parameters were represented in terms of I; the ionization potential and A; the electron affinity [44]. All the previous terms were calculated as reported [46,47].

The charge transfer examined the reactivity index term, “ΔN_max_”, which measured the stabilization energy when the system gains an additional charge from the environment (Table 1). The total binding energy measured the maximal electron transfer in the ground and valance states and represented in terms: “ΔE_ma_^GS^” and “ΔE_max_^VS^ (Table 1). This work examined the electrophiles and nucleophiles based on both soft and hard terms. The soft nucleophiles and hard electrophiles related to ΔG, that can measure the stability index. The molecule with low ΔG has high polarizability, softness, chemical reactivity, and nucleophilicity (thereby easily offering electrons to kinase) and vice versa. Table 1 showed the low softness values (~0.250 −0.296 eV.) for the (**3**–**6**, **9**, **11**) compounds, that may join to form a high degree of reactivity to the biological environment.

All (**3**–**6**, **9**, and **11**) compounds showed a similar value via (ω^+^), (μ^+^), and (ω^+^) and were arranged as, bithiazolidinones 11 > free acid 6 > thioester 9 > acetyl 5 > potassium salt 3 > 4 (Table 1). The previous order reversed for electron donating behaviors (μ^−^, ω^−^). The data in (Table 1) proved that the molecular skeleton bearing di-thiazolidine-2,4-dione rings **11**, thioester **9**, and carboxyl group **6**, can improve the obtaining of an electron from the kinase. Compounds **6**, **9**, **11** have the highest range of net electrophilicity (ω ± = 9.847 to 9.58 eV.). Thus, these compounds have promising electrophilicity, that may enhance the attacking chance over a polar active site of the receptor. In addition, our studies [48,49] showed a direct relationship between antioxidant ability and IP, with the former increasing as the latter’s value decreases. The antioxidant molecule can convert the electron into a stable cationic free radical that promotes healthier radical scavenging via a one-electron transfer mechanism [48]. As reported [50], the antioxidant ability increased with decreasing IP value. Thus, the investigated compounds **3**–**6**, **9**, **11** displayed promising antioxidant activity. Based on the above data, all of the calculated descriptors for **3**–**6**, **9**, **11** were harmonized with the reported value for several biomaterials [51,52].

#### 3.2.2. Molecular Electrostatic Potential (MEP)

**Figure 3:**
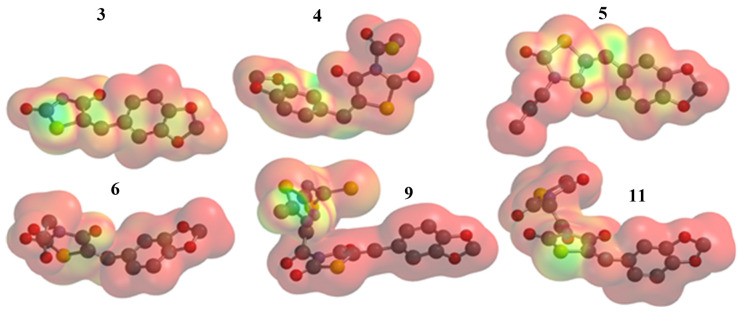
Plotted MEP of synthesized compounds (**3**–**6**, **9**, and **11**).

The DFT plotted the molecular electrostatic potential (MEP) in (Figure 3) for the (**3**–**6**, **9**, **11**) compounds. The MEP figured a balance between the repulsive interaction attributed to nucleophilic ability, and attractive interactions related to electrophilic reactivity. The orange, yellow, and red areas depicted negative power (great electron density area). The blue shift figured a positive potential, while green color represented an intermediate potential ability. The MEPs showed a high level of electron density (showed in red color) covered over most of the backbone of the **3**–**5**, **9**, and **11** compounds. The red regions indicated electrophilic potency. We explained the variation in shading for the MEPs by the diversity of the electrostatic potential values. That was attributed to substrate → receptor identification and caused the electrostatic interactions ability to expand [53].

### 3.3. Docking Study

The molecular docking study targeted a PPAR-γ and α-amylase to examine a mode of regulation action for the investigated compounds. The ligand–protein interaction behavior was estimated based on the gold score function as implemented in MOE.2019 [54]. The probe compounds were successfully docked into the reported active sites (CYS 285, GLu233, HIS 449, SER299, HIS323, CYS285,ASP300, Arg195) and (Asp300, Glu233 and Asp197) for (PPAR-γ, PDB: 2PRG [55]) and (α-amylase, PDB:2QV4 [56]), respectively.

The **3**–**6**, **9**, and **11** ligands were complexed successfully over the active sites, then minimized using the (MMFF94) force field. The highest MOE scoring function with the lowest RMSD was applied to evaluate the binding efficiency BE for the investigated compounds (Table 2).

We calculated the inhibition constant (Ki) for further validation of the BE [57]. The bioactivity factor was also examined as the ligand-efficiency (LE) and fit-quality (FQ) [58]. All the synthesized compounds showed a BE higher than reference inhibitors (−7.86 and −5.59 kcal/mol) for PPAR-γ and α-amylase respectively (Table 2).

**Figure 4:**
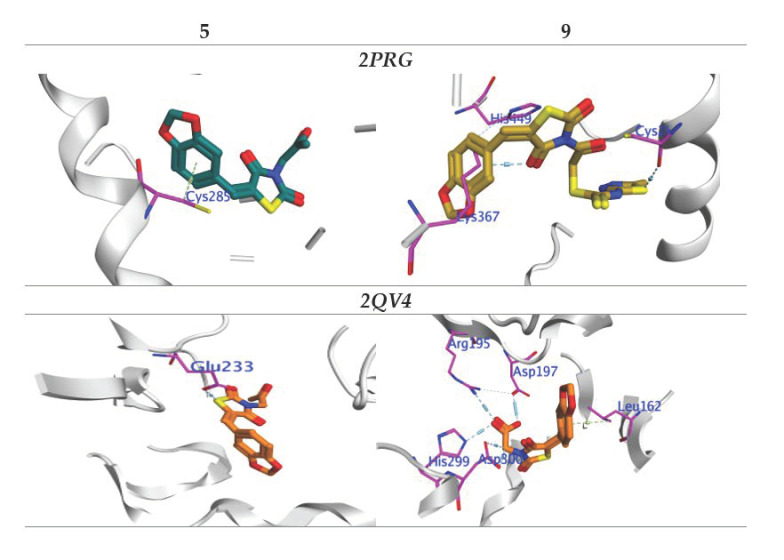
Interaction between ligands **5** and **9** with 2PRG and 2QV4 binding sites, which blue lines represented hydrogen bonding interaction.

In PPAR-γ: The compounds (**5** and **9**) exhibited the highest BE (−11.85 and −11.40 kcal/mol), with promising Ki (1.61 and1.65 μM), respectively (Table 2). The BE for the remaining hybrids were arranged according to **4** < **11** < **6** < **3**. All of the compounds showed a normal range of bioactivity parameters, such as Ki, LE, and FQ [59]. The **5** and **9** interacted into a binding site in an identical manner, through an important hydrogen bond with Cys285, which was arranged in a perpendicular mode with their compounds (Figure 4).

In α-amylase: The (**5** and **9**) also showed the most BE (−6.74 and −7.66 kcal/mol), with the lowest inhibition constant Ki (2.05 and 2.17 μM), respectively (Table 2). The BE for other members is organized as **4** < **6** < **3** < **11**. All of the compounds showed a normal range of bioactivity parameters (Ki, LE, and FQ) [59]. Compound **5** formed H-bond with Glu233 residue, while compound **9** made H-bond with important amino acids Arg.195, ASP197, His.299, and Asp300, and also formed a π–π interaction with backbone residue Leu.162 (Figure 4).

### 3.4. Biological Study

#### 3.4.1. The In Vitro α-Amylase Inhibitory Profile

**Figure 5:**
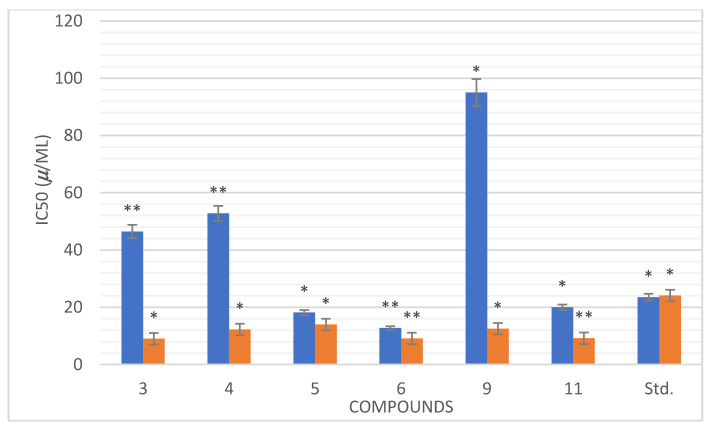
IC_50_ in (μg/mL) for the tested compounds (**3**–**6**, **9**, and **11**) compared to ascorbic and acarbose, respectively, as standard drugs for DPPH radical (orange) and α-amylase enzyme (blue), respectively. Data analyzed by one way ANOVA followed by LSD test and expressed as mean ± SEM from five observations; * represents change as compared to control; ** indicates *p* < 0.01 and * shows *p* < 0.05.

This study examined the inhibitory potential for the (**3**–**6**, **9**, and **11)** compounds against α-amylase enzyme. We applied six different concentrations (5,10,15,20,30, and 40) µg/mL for this test. These concentrations were plotted via the inhibition percentage to determine the IC_50_ (the concentration required to inhibit 50% of the enzyme) using the non-linear regression method. All of the tested compounds showed a marked inhibitory potential value about IC_50_ = 9.06 to 13.98 µg/mL compared to the “STD” standard drug (Acarbose) IC_50_ = 24.1 µg/mL (Figure 5). The compounds **5**, **6**, and **11** showed a higher inhibitory action than acarbose, with IC_50_ = 18.02, 10.26, and 20.01 µg/mL. The **3** and **4** displayed moderate potency, with IC_50_ = (43.23 and 52.98 µg/mL), with significance (*p* < 0.05) when compared to the positive control. Figure 5 shows that the parent compound with these fragments [acetyl (5), carboxy (**6**) and extra thiazaldinone (**11**)] in the corner increased the amount of activity. On the other hand, the inhibition potency decreased with the introduction of acetyl, K salt **(3)**, chloracetyl (**4**), and thiadiazole (**9**) to the molecular skeleton. We can explain the highest efficiency due to the increasing polarity feature in (**5** and **6**) and hydrophilicity in **11**, which can interact with the hydrophobic part of the active site.

#### 3.4.2. In Vitro Anti-Oxidant Activity

We examined the antioxidant activity of the (**3**–**6**, **9**, and **11**) hybrids using the stable DPPH assay [37]. The antioxidant result was represented as IC_50_ (the concentration is essential for 50% reduction in the power of H-donation or radical scavenging). We estimated IC_50_ based on different concentrations (0.16, 0.8, 4, 20, 40, 200, and 1000 µg/mL), as shown in Figure 5. All of the compounds showed a higher potency than Vitamin C and arranged **Vit.C** < **9** < **5** < **4** < **3** < **11** < **6**. Compounds **6** and **11** with the terminal carboxy and bi-thiazaldinone groups, respectively, showed the highest scavenging effect (IC_50_ = 10.78 and 11.16 μg/mL). The increasing scavenging activity may be due to the stabilization of the lone pair in the conjugated system through the presence of the terminal carbonyl and thiazolidinone groups.

#### 3.4.3. The In Vivo Regulation of Blood Glucose Level

This study used an alloxan-induced diabetic rat model to test the anti-hyperglycemic action of substances (**3**–**6**, **9**, **11**). Diabetes was successfully induced in the rats under investigation, as verified by raised their blood glucose level “BGL” compared with the normal control rats. The BGL was monitored for each tested compound at three different doses (50, 100, and 250 μ/Kg) and for five experimental period durations (0, 2, 4, 15, 30 days).

**Figure 6:**
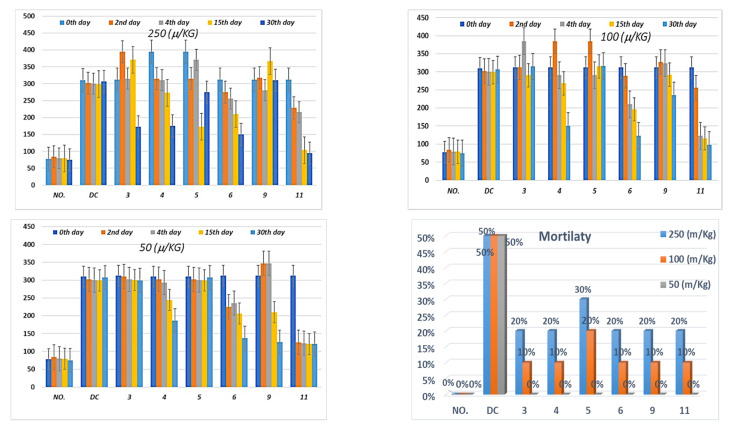
Antidiabetic effect and percent of mortality in three different concentration 50, 100, and 250 (μ/Kg) at 0th day (pre-drug values), 2nd and 4th day (post-drug values) for each group of compounds (**3**–**6**, **9**, **11**) compared to diabetic-control “DC” and normal control “NO”. Data analyzed by one way ANOVA followed by LSD test and expressed as mean ± SEM from five observations.

Compared with the control at 250 μ/Kg (Figure 6): all tested compounds (**3**–**6**, **9** and **11**) exhibited insignificant decrease in BGL compared to the normal group at pre-treatments. Compounds **5** and **11** showed promising decreases in BGL at the 15th and 30th treatments. Conversely, only compounds **6** and **11** at the 30th time duration of treatment showed a significant reduction in glucose level. At 100 μ/Kg in comparison with DC (Figure 6), the compounds (**4**, **6**, and **9**) exhibited moderate decreases in BGL at the 4th and 15th time durations, while compound **11** showed a significant reduction in glucose level at the 4th, 15th and 30th time durations. The results obtained from (Figure 6) showed a promising reduction in BGL at 50 μ/Kg with (*p* < 0.05) for the treatment of compound **11** group without mortality during all the experiment periods compared to the DC group. The **4**, **6** and **9** groups figured a moderate reduction in BGL value at 50 μ/Kg during most of the experimental periods at (4th,15th, and 30th). At 50 μ/Kg, we observed no mortality during any of the experiment periods for all tested compounds.

#### 3.4.4. Lipid Profile

**Figure 7:**
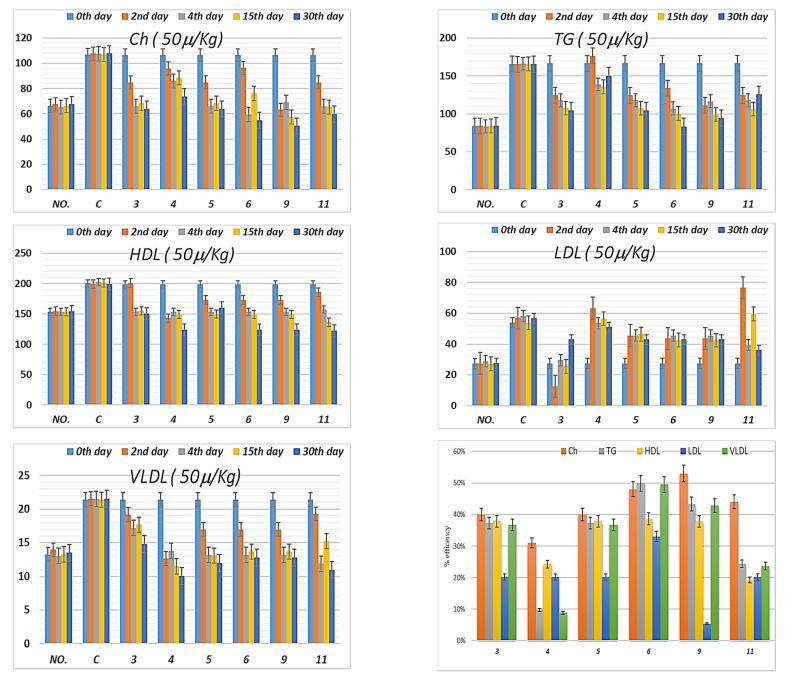
The level of Ch (cholesterol), TG (triglyceride), HDL (high density lipoprotein), LDL (low density lipoprotein) and VLDL (very low density lipoprotein) in serum of hyperglycemic loaded rats. 50 (μ/Kg) on 0th day (pre-drug values), 2nd and 4th day (Post-drug values) for each group of compounds (**3**–**6**, **9**, and **11**) and control. Data analyzed by one way ANOVA followed by LSD test and expressed as mean ± SEM from five observations.

A serious side effect related to diabetes is Hyperlipidemia [60,61]. Insulin deficiency promotes the adipocytes to generate a fatty acid, hepatic phospholipids and cholesterol [62]. Thus, we measured the levels of (cholesterol “CH”, triglyceride “TG”, high, low and very-low- and high/low and very low density lipoproteins “HDL, LDL and VLDL”) in serum for alloxan-loaded diabetes. Figure 7 showed the efficiency percentage for these parameters for the tested compounds and the normal control. All of the lipid parameters for compounds (**3**–**6**, **9**, and **11**) have (*p* < 0.05) compared to the normal control group (Figure 7). The serum CH. Level decreased by 40%, 32%, 40%, 48%, 52%, and 43% for **3**–**6**, **9**, and **11**, respectively, compared to the diabetic control group (Figure 7). The compounds **3**–**6**, **9**, and **11** shrank CH level nearly to a normal level of the control animals, on the 4th and 15th days. The **3**, **5**, **6**, **9**, and **11** groups showed a lower CH level than the normal animals, at the 30th day (Figure 7).

On the contrary, the triglyceride level (TG) increased above the normal value when treated by the tested compounds. The TG level of alloxan-induced diabetes exceeded by 50.09% higher than the normal control animals throughout the experimental period (0th–30th days). The groups of compounds **3**–**6**, **9**, and **11** failed to regulate the TG level to the normal level, except for group **6**, whose TG level decreased to the normal level (at the 30th day). All group members displayed lower TG level than the alloxan-induced diabetes control group, with a decreasing range of 9.69%–49.92% at the 30th day (Figure 7).

The HDL serum level for all of the groups improved compared to the normal control group at 15th days. All of the compounds significantly reduced the HDL value of infected rats by around 18% to 38%. The tested members successfully drove the HDL level of the infected rats to almost a normal HDL value during periods of the experiment (4th and 30th days).

In all periods of the experiment, the serum LDL level for all group members increased compared to that of the normal rats, with an LDL value range 15.12–78.25 μ/kg. Compound **3** can regulate the LDL value to the normal value on the 2nd and 15th days, but failed to do so on the 30th. Compared with the infected animals, all the compounds monitored LDL level lower than these rats for all periods, except (**4**) on 2nd and (**11**) 2nd and 15th days, with a reduction range of 5.35% to 35.58% (Figure 7). Molecule **4** displayed a VLDL level of serum below the normal value for all experimental periods compared to the normal rats. The **5**, **6**, and **9** groups regulated the VLDL level to the normal level on the 4th, 15th, and 30th days, while on 2nd day these compounds suffered from regulation the VLDL to normal level. Compound **11** regulated only the VLDL of infected rats to normal values on the 4th and 30th day. The group of **3** compound failed to reach to control value for VLDL at the 2nd to 15th days while, on 30th day, this group almost reached to the normal VLDL level.

### 3.5. In Silico Pharmacokinetic Profile

The oral bioavailability behaves as a vital role in the improvement of a therapeutic molecule. The MOE, and admet-SAR model simulated all descriptors. The obtained result is disclosed in (Table 3). The drug likeness was studied according to the Lipinski rule [63]. Table 3 showed that the tested compounds obeyed to their rule without violation, with a high absorption percentage (%ABS) in the range (66.7%–73.83%) [64]. The topological polar surface area (TPSA) for the tested compounds appeared promising, which was linked to good oral bioavailability properties [64].

**Figure 8:**
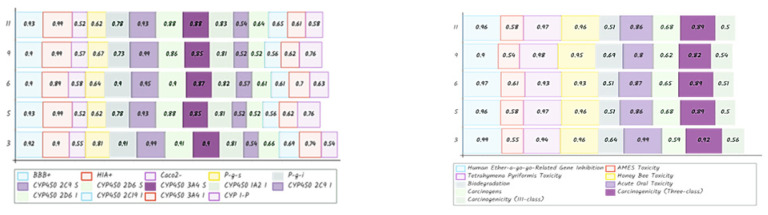
Absorption, distribution, and toxicity of pharmacokinetic parameters derived from the ADME-Tox for ligands (**3**, **5**, **6**, **9** and **11**).

We investigated the carcinogenic effect (Figure 8), by comparing our structures with 981 various carcinogenic structures gained from the “Carcinogenic Potency Database (CPDB)”. The result exhibited that there is no carcinogenicity effect with the range of values (~0.6–0.9 mg/kg body wt./day). The synthesized compounds have a good oral bioavailability, high ability BBB transport, and no marked health effects observed for the rodent toxicity profiles.

### 3.6. SAR Profile

The antihyperglycemic compounds, based on the thiazolidinedione scaffold were synthesized and characterized. The FMOs and global reactivity data indicated that the intramolecular charge transfer takes place between the thiazolidine-2,4-dione and benzodioxole corner in two directions. The MEP for these compounds represented that they have promising electrophilicity, that may enhance the attacking chance against the polar active site of the receptor. The molecular docking into the PPAR-γ and α-amylase showed the high binding affinity for the synthesized compounds, and may be a suitable regulator for both kinases. The in vitro inhibition potency via the α-amylase was evaluated and represented that the parent compound bearing these corners acetyl (**5**), carboxyl (**6**), and thiazaldinone (**11**) is more efficient than acarbose. This efficacy decreased in the presence of K salt (**3**), acetyl chloride (**4**), and thiadiazole (**11**) in the molecular skeleton. All hybrids (**3**–**6**, **9**, and **11**) have a higher scavenging ability than Vitamin C. The molecular skeleton with K salt (**3**), carboxy (**6**), and bi-thiazaldinone (**11**) has a healthy antioxidant activity compared to the other members. The bi-thiazolidinone in (**11**) hybrid was the most potent regarding the regulation of BGL, Ch, TG, HDL, LDL, and VLDL levels at different concentrations at the 4th, 15th, and 30th days. All of the compounds enhanced the antihyperlipidemic effect compared to the DC. The antihyperglycemic efficiency improved with the introduction of an extra thiazolidinedione ring (**11**), or free carboxylic acid fragment (**6**) on the 4th, 15th, and 30th days compared to the DC. The in silico ADMET of the tested compounds showed a good oral bioavailability and high BBB transport ability. The rodent toxicity profile showed that no marked carcinogenic and non-healthy effect was observed for the synthesized compounds.

## 4. Conclusions

The new thiazolidinediones were synthesized and characterized. The FMOs and global reactivity data indicated that an intramolecular charge transfer occurs in two directions between the benzodioxole and thiazolidine-2,4-dione corners. The MEPs represented that they have promising electrophilicity, that may have enhanced the attacking chance over the polar active site of the receptor. The molecular docking simulation into the PPAR-γ and α-amylase displayed that the synthesized compounds may be a suitable regulator for both kinases. The inhibition potency via the α-amylase in vitro showed that the compounds **5**, **6**, and **11** displayed the highest efficacy against acarbose and other compounds. Conversely, all of the compounds had a higher ability to scavenge radicals compared to Vitamin C. All of the compounds exhibited insignificant BGL pre and post the experimental periods compared to NO and DC, while, after the 30th treatments, they successfully decreased the BGL for all concentrations. None of the compounds exhibited any mortality at 50 μg/mL for all experiment durations. Moreover, all the compounds at 50 μg/Kg enhanced the antihyperlipidemic effect compared to the normal level. ADMET profile in silico for these compounds displayed a promising oral bioavailability and BBB transport ability without any discernible carcinogenic or non-healthy effects in rodents.

## Figures and Tables

**Table 1:** Calculated energetic reactivity parameters for compounds (**3**, **5–12**) at DFT with B3LYP\6-311G * Basics set.

	3	4	5	6	9	11		3	4	5	6	9	11
HOMO	−8.18	−6.839	−8.56	−8.93	−8.81	−9.23	µ^+^	−2.99	−1.79	−2.972	−3.345	−3.245	−3.35
LUMO	−1.26	−0.108	−1.11	−1.35	−1.39	−1.39	µ^−^	−6.45	−5.16	−6.697	−7.035	−6.955	−7.027
ΔG	−6.92	−6.731	−7.45	−7.58	−7.42	−7.84	ω^−^	6.11	3.95	6.21	6.529	6.519	6.741
IP	8.18	6.839	9.23	8.93	8.81	8.56	ω^+^	2.786	1.37	2.672	3.011	3.041	3.106
A	1.26	0.108	1.11	1.35	1.39	1.39	ω^±^	8.798	5.32	8.693	9.58	9.56	9.847
η	3.46	3.365	3.725	3.79	3.71	3.92	ΔN_max_	−0.682	1.03	−0.648	−0.678	−0.687	−0.677
S	0.289	0.297	0.268	0.263	0.269	0.255	${\Delta E}_{\max}^{\mathrm{GS}}$	2.728	−1.53	2.595	2.712	2.06	2.709
χ	−4.72	−3.473	−4.835	−5.14	−5.1	−5.31	${\Delta E}_{\max}^{\mathrm{VS}}$	−0.682	−0.84	−0.648	−0.678	−0.687	−0.677
ω_i_	3.219	3.453	3.137	3.485	3.505	3.596							

**Table 2:** Docking energy scores (kcal/mol) derived from the MOE for (**3**–**6**, **9**, and **11**).

Cpd.	Ref.Inhibtor	3	4	5	6	9	11
	PPAR-γ(PDB: 2PRG)						
ΔΕ	−7.86	−9.80	−6.48	−11.85	−9.14	−11.40	−9.01
RMSD	1.95	1.60	1.50	1.33	1.79	1.77	1.89
E__place_	−47.36	−112.41	−39.25	−37.56	−59.86	−89.46	−118.45
E._Int._	−35.63	−30.39	−33.70	−32.85	−34.63	−38.27	−33.03
E._H.B._	−0.25	−1.33	0.29	−0.65	−0.12	0.37	−0.23
L.E	4.03	6.76	4.34	8.88	5.11	6.46	4.76
Ki(μM)	1.39	1.70	2.21	1.61	1.87	1.65	1.88
LE_scale_	2.67	2.84	2.90	3.00	2.74	2.75	2.69
FQ	1.36	3.92	1.44	5.88	2.37	3.70	2.07
	α-amylase, (PDB:2QV4)						
ΔΕ	−5.59	−6.23	−6.50	−6.74	−6.14	−7.66	−6.33
RMSD	1.79	0.93	1.33	1.95	1.95	1.19	1.37
E__place_	−26.39	−43.10	−62.93	−23.29	15.12	−113.81	−37.51
E._Int._	−32.26	−24.52	−19.04	−24.48	−15.75	−26.46	−18.00
E._H.B._	−17.58	−15.46	−10.35	−11.87	−10.07	−11.36	−10.01
L.E	3.12	6.67	4.89	3.46	3.15	6.44	4.61
Ki(μM)	1.14	2.25	2.21	2.17	2.27	2.05	2.24
LE_scale_	−2.74	−3.31	−3.00	−2.67	−2.67	−3.10	−2.97
FQ	0.38	3.36	1.89	0.79	0.48	3.34	1.64

ΔE: free binding energy of ligand-pose which derived from gold tool, RMSD; the root mean square deviation of the docking pose compared to the co-crystal ligand position, E__place_.: free binding energy of ligand-receptor. E_.Int._: binding-affinity energy of ligand-receptor, E._H.B._: energy H-bonding between protein and ligand.

**Table 3:** Drug likeness parameters and derived for **3**, **5**, **6**, **9**, and **11** ligands.

Cpd.	3	5	6	9	11
HBD	1	2	4	3	2
HBA	8	9	8	7	8
Log.P	2.83	3.21	2.25	2.42	2.74
Log.S	−5.53	−6.13	−5.73	−4.26	−5.11
V	0	0	0	0	0
TPSA	105.5	132.48	157.05	112.93	116.5
%ABS	72.6	73.83	66.7	70.03	68.8
Mutagenic	none	none	none	low	none
Tumorigenic	none	none	none	high	none
Reproductive Effective	none	none	none	high	none
Irritant	none	none	none	high	none

## Data Availability

Data Available in “MDPI Research Data Policies” at https://www.mdpi.com/ethics (accessed on 25 October 2021).

## Supplementary Materials

The following are available online at https://www.mdpi.com/article/10.3390/biomedicines10010024/s1, Figure S1: superimpose for the most active compounds.
